# Primary lymphoma of the kidney: Pathology experience from 2 tertiary hospitals in Western Saudi Arabia

**DOI:** 10.15537/smj.2023.44.1.20220772

**Published:** 2023-01

**Authors:** Jaudah A. Al-Maghrabi

**Affiliations:** *From the Department of Pathology, Faculty of Medicine, King Abdulaziz University, Jeddah, Kingdom of Saudi Arabia, and from the Department of Pathology, King Faisal Specialist Hospital and Research center, Jeddah, Kingdom of Saudi Arabia.*

**Keywords:** lymphomas, kidneys, Saudi Arabia

## Abstract

**Objectives:**

To document the clinicopathological features of primary renal lymphoma (PRL) at 2 tertiary hospitals in the western region of the Kingdom of Saudi Arabia.

**Methods:**

Data were retrieved from all PRLs that were diagnosed at King Abdulaziz University Hospital and King Faisal Specialist Hospital and Research Centre, Jeddah, Saudi Arabia, between January 2002 and April 2022. Histopathological and immunohistochemical slides were reviewed, and additional immunohistochemistry stains were carried out in selected cases. Follow-up data were also collected.

**Results:**

There were 17 identified cases of PRL. The age of the patients ranged from 4-76 years (median: 50.5 years and mean: 46.8 years), 11 (64.7%) were males, and 6 (35.3%) were females. There were 12 cases of diffuse large B-cell lymphoma, 3 cases of Burkitt’s lymphoma, and 2 cases of post-transplant lymphoproliferative disorder. The median follow-up duration was 22 months. The one-year overall survival rate was 71% and the 2-year overall survival rate was 43% during follow-up.

**Conclusion:**

Primary renal lymphomas in Saudi patients are more common in males and seen in a relatively younger age group compared to the available worldwide data. The diagnosis of PRL is important to avoid tumor dissemination and unnecessary nephrectomy. Diffuse large B-cell lymphoma is the most common pathological type and non-germinal center B-cell is the most common subtype.

**L**ymphoid neoplasms that arise primarily in the kidneys are rare. These lymphomas are critical to diagnose and differentiate from non-hematopoietic renal malignancies because of the different management. The definition of primary versus secondary lymphoma of extranodal sites continues to be controversial. Primary renal lymphoma (PRL) is defined by some authors as lymphoma that involves the kidneys without extensive node disease, and while others define it as all presentations of non-Hodgkin’s lymphoma (NHL) that originates at kidney site, even in the presence of disseminated disease, if the renal component is clinically dominant.

Secondary renal involvement in NHL is relatively common in advanced stages and can be seen in up to 30-60% of NHL patients. However, PRL is extremely rare and comprises less than 1% of all renal masses and less than 1% of extranodal lymphomas.^1-6^ Primary renal lymphoma is a distinct clinicopathological entity with highly aggressive behavior, and 50% of the cases also exhibit extrarenal involvement.^6,7^ Utilizing the Surveillance, Epidemiology, and End Result (SEER) database, Taneja et al^2^ and Chen et al^4^ showed that diffuse large B-cell lymphoma (DLBCL) is the most common histopathological subtype. Other very rare histological types have been described in the literature, including Hodgkin’s lymphoma.^8^

Little is known on the pattern of PRL in Saudi Arabia. The information is limited to a few case reports.^6,9,10^ Therefore, the aim of this study was to review all the cases of PRL diagnosed at 2 major tertiary hospitals in the western region of Saudi Arabia.

## Methods

This retrospective study was carried out at King Abdulaziz University Hospital and King Faisal Specialist Hospital and Research Centre, Jeddah, Saudi Arabia, which are 2 main referral hospitals in the western region of Saudi Arabia. Inclusion criteria included all cases diagnosed as kidney lymphoma between January 2002 and April 2022. The project started in January 2022 and was completed in August 2022. Exclusion criteria included cases with no available pathology slides and paraffin blocks. The definition proposed by Krol et al^11^ was used to define primary extranodal NHL, which includes all patients who present with NHL originating at the kidney, even in the presence of disseminated disease, if the kidney component is clinically dominant.

The collected clinical data included age at presentation, gender, clinical features, and treatment. The immunohistochemistry slides were reviewed, and more immunohistochemistry markers were added in selected cases. The minimum immunohistochemistry panel included CD45, CD20, CD3, BCL-2, BCL-6, CD10, MUM-1, and KI-67. Additional panels were added in selected cases and included CD21, CD23, CD30, CD79a, CD38, CD138, Fascin, PAX-5, CD15, Epstein-Barr virus (EBV), and pankeratin. The additional panel was carried out to confirm subtype, to rule out Hodgkin lymphoma and viral inclusions. Histopathological classification of lymphomas was according to the 2017 World Health Organization (WHO) criteria.^12^ Diffuse large B-cell lymphomas were subclassified according to Hans algorithm to germinal center B-cells (GCB) and non-GCB which depends on the pattern of immunohistochemical expression for CD10, MUM-1, and BCL-6.^13^ The study was approved by the Research Committee of the Biomedical Ethics Unit at King Abdulaziz University, Jeddah, Saudi Arabia (reference no.: 34-22). The study was carried out according to the principles of Helsinki Declaration. A review of morphology and additional immunohistochemistry markers allowed the re-classification of older cases into currently accepted diagnostic categories.

### Statistical analysis

Chi-square test was used to assess the difference in bilaterality among different types of lymphoma.

## Results

There were 17 identified cases of PRL. Clinicopathological data is summarized in Table 1. The age of the patients ranged between 4-76 years (median: 50.5 years and mean: 46.8 years). All cases were seen among adult patients except for one pediatric case. There were 11 (64.7%) males and 6 (35.3%) females. The clinical manifestation included renal masses (11 cases), flank or pelvic pain (7 cases), hematuria (6 cases), renal function impairment (5 cases), weight loss (2 case), epistaxis (one case), and vomiting (one case). There were 14 (82.4%) unilateral cases and 3 (17.6%) bilateral cases. Two of the bilateral lymphomas were Burkitt’s lymphoma, and the other was DLBCL. Bilateral involvement is rare and seen more in Burkitt’s lymphoma than DLBCL (*p*=0.024).

**Table 1:** - Summary of the primary renal lymphoma cases from 2 tertiary hospitals in the Western regions of Saudi Arabia.

Patient no.	Age/gender	Specimen	Site	Clinical presentation	Diagnosis	Bone marrow	Radiological findings
1	50/M	Biopsy	Left kid	Palpable mass and hematuria	DLBCL	-ve	CT: hypodense lesion in the left kid. The spleen and liver are normal. No significantly enlarged LNs.
2	56/M	Biopsy	Right kid	Hematuria, flank pain	DLBCL	-ve	US: hypoechoic tumor with enlarged retrocaval lymph nodes. The spleen and liver are normal.
3	59/F	Biopsy, referral	Right kid	Hematuria, weight loss	DLBCL	Not carried out	CT: hypodense lesion in the right kid (3.4x3.2x3 cm). No enlarged lymph nodes. Normal spleen and liver
4	61/M	Biopsy, referral	Left kid	Palpable mass and hematuria	DLBCL	-ve	CT: enhancing mass involving left kid There are enlarged lymph nodes in paracaval region. The spleen and liver are normal.
5	63/F	Biopsy	Left kid	Flank pain	DLBCL	Not available	Not available
6	23/M	Biopsy	Left kid	Abdominopelvic pain	Burkitt’s lymphoma	-ve	CT: multiple hypodense lesions involving left kid. No enlarged lymph nodes. Normal spleen and liver
7	24/M	Biopsy	Right kid	Flank pain and mild renal impairment	DLBCL	-ve	US: swollen right kid with paraaortic lymph node enlargement.
8	39/M	Biopsy	Kid, bilateral	Chronic renal failure, bilaterally enlarged kid suggestive of glomerulonephritis	Burkitt’s lymphoma	-ve	CT: both kids enlarged with multiple hypodense lesions. No enlarged lymph nodes. Normal spleen and liver
9	53/M	Biopsy	Right kid	Hematuria, loss weight for 2 months, known hypothyroid and chronic renal failure	DLBCL	-ve	CT: hypodense lesion in the right kid. The spleen and liver are normal. No significantly enlarged LNs.
10	4/M	Biopsy	Kid, bilateral	Bilateral renal enlargement with hepatomegaly	Burkitt’s lymphoma	-ve	CT: both kids are enlarged with no defined focal lesions. There are a few periaortic lymph nodes. Enlarged spleen with no focal lesion. Normal liver. CT of orbits: this is involvement of both orbits and both maxillary sinuses.
11	36/F	Biopsy	Kid, bilateral	Epistaxis, abdominal pain, vomiting, and bilateral enlarged kid	DLBCL	-ve	CT: significant intraabdominal lymphomatous infiltrative disease involving the abdominal organs namely both kids, liver, gallbladder, pancreas, adrenals, and intraabdominal and pelvic lymph nodes.
12	52/F	Biopsy	Left kid	Left renal mass	DLBCL	-ve	CT: perinephric soft tissue lesion is seen involving the right kid (2.8x0.8 cm). No enlarged lymph nodes. The left kid is atrophic. Liver, spleen, and pancreas adrenal glands are normal.
13	76/F	Biopsy	Left kid	Left renal mass, hematuria	DLBCL	-ve	CT: hypodense lesion in the left kid (3.6x4.3x3.4 cm). Similar lesion infiltrating focally the mid-portion of descending colon measuring (2.0x3.5 cm). There are multifocal hypodense splenic lesions. The pelvic organs are unremarkable.
14	41/M	Radical nephrectomy	Right kid	Right kid mass with enlarged paraaortic lymph node	DLBCL	-ve	CT: hypodense lesion with enlarged lymph nodes in paracaval region just below the level of renal vein measuring 2.3 cm.
15	53/F	Biopsy	Right kid	Right renal mass and flank pain	DLBCL	-ve	CT: enhanced lesion in the right kid (4.6x3.9 cm). It shows slightly more extension towards the renal hilum. The left kid, liver, and spleen are unremarkable.
16	61/M	Biopsy	Transplanted kid	Renal mass, flank pain	PTLD, monomorphic, monoclonal, and EBV associated-B cell/DLBCL	Not carried out	CT: large enhancing retroperitoneal mass encasing and involving transplanted kid. There are multiple retroperitoneal, retrocaval, and bilateral inguinal lymph node enlargement.
17	44/M	Biopsy	Transplanted kid	Renal mass	PTLD, monomorphic, monoclonal, and EBV associated-B cell/DLBCL	-ve	CT: enhancing mass involving transplanted kid associated with enlarged retrocaval lymph nodes.

Kid: kidney, M: male, F: female, DLBCL: diffuse large B-cell lymphoma, PTLD: post-transplant lymphoproliferative disorder, EBV: Epstein-Barr virus, -ve: negative, LNs: lymphoid neoplasms, CT: computed tomography, US: ultrasound

Radiological evaluation by computed tomography (CT) scan was available in 14 cases, which revealed one or more renal hypodense lesions as the most common feature, both with local enlarged lymph nodes and without them. In 5 patients there was a solitary mass without associated lymph node enlargement. In 2 patients there was significant extrarenal involvement. In one of these patients, CT showed significant intraabdominal lymphomatous infiltrative disease involving the abdominal organs (both kidneys, the liver, gallbladder, pancreas, adrenals, and intra-abdominal and pelvic lymph nodes. In the other patient, CT showed a hypodense lesion in the left side and similar lesions focally infiltrating the mid-portion of the descending colon associated with multifocal hypodense splenic lesions. In 2 other cases, only ultrasound (US) studies were available, and both had hypoechoic tumors. In one case, no radiology results could be retrieved.

There were 16 patients who were diagnosed based on renal biopsy materials, and one involved a radical nephrectomy specimen that was referred to our hospital for pathological consultation. Biopsies were carried out for those patients because of atypical clinical or radiological appearance that was not typical for renal cell carcinoma.

Pathological examination revealed that all cases were non-Hodgkin’s B-cell lymphoma. There were 12 (70.6%) cases of DLBCL, 3 (17.6%) cases of Burkitt’s lymphoma, and 2 (11.8%) cases of post-transplant lymphoproliferative disorder (PTLD). All cases of DLBCL showed morphological features of DLBCL, not otherwise specified (Figure 1A-D). The proliferating cells were large with vesicular nuclei and prominent nucleoli. Tumor cells infiltered and destroyed the renal parenchyma. They expressed a typical immunoprofile with positive staining for CD45 and CD20 with negative results for CD3. The Ki-67 range was 50-85%. Two (16.7%) of the DLBCLs were further subclassified to be GCB subtype, and 8 (66.7%) were subclassified into the non-GCB subtype. In the other 2 cases, no material was available to carry out immunohistochemistry study required for subtyping. The 3 cases of Burkitt’s lymphoma revealed a “starry-sky” appearance with medium-sized lymphocytes. Tumor cells had scant to moderate cytoplasm, round nuclei, and multiple coarse nucleoli (Figure 1E-H). Immunohistochemistry staining in these tumors showed a typical immunoprofile of Burkitt’s lymphoma with positive staining for CD45, CD20, CD10, and BCL-6 and a negative result for BCL-2. All showed almost 100% Ki-67 staining.

**Figure 1:**
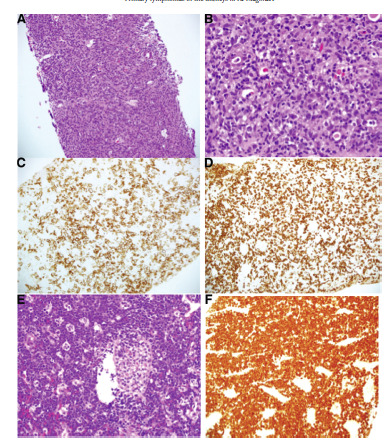
- Lymphoma of the kidney. **A**) Section of renal mass biopsy showing diffuse large B-cell lymphoma (DLBL) composed of diffuse infiltration of large lymphoid cells ddestroying renal parenchyma (hematoxylin and eosin, 200×). **B**) Higher power of the same case; reveal large lymphoma cells surrounding renal tubules (hematoxylin and eosin, 400×). **C**) DLBL expressing CD20 (immunohistochemistry stain, 200×). **D**) DLBL with negative staining for CD3, which highlighted reactive T-cells (immunohistochemistry stain, 200×). E) Section of renal mass biopsy showing Burkitt’s lymphoma composed of monomorphic intermediate sized lymphoid cells with starry sky appearance showing tangible body macrophages and surrounding glomerulus (hematoxylin and eosin, 400×). F) Burkitt’s lymphoma expressing Ki-67 in almost 100% of tumor cells (immunohistochemistry stain, 400×).

**Figure 1:**
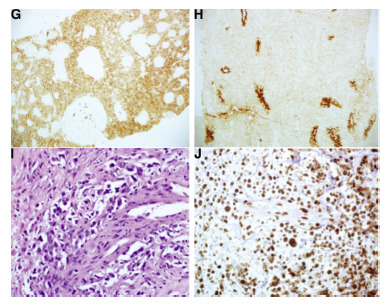
- Lymphoma of the kidney. **G**) Burkitt’s lymphoma expressing CD20 (immunohistochemistry stain, 200×). **H**) Burkitt’s lymphoma expressing CD10 (immunohistochemistry stain, 200×). **I**) Post-transplant lymphoproliferative disorder composed of large atypical lymphoid cells with areas of necrosis (not shown). (Immunohistochemistry stain, 400×). **J**) Post-transplant lymphoproliferative disorder, Epstein-Barr virus stain showing positive staining in proliferating cells (immunohistochemistry stain, 400×).

One of the PTLDs developed 17 months after transplant in a 44-year-old male, and the other developed at 14 months after a transplant in a 61-year-old male. Post-transplant lymphoproliferative disorder cases were a monomorphic, monoclonal B-cell, EBV-positive type (Figure 1I&J). Immunohistochemistry stains for cytomegalovirus (CMV) were negative in both PTLD cases. No low-grade lymphoma, Hodgkin’s, or T-cell lymphoma was identified. Clinical follow-up data were available in 14 patients (10 DLBCL, 2 Burkitt’s lymphomas, and 2 PTLD). Nine patients with DLBCL received standard therapy with rituximab, cyclophosphamide, doxorubicin, vincristine, and prednisolone (R-CHOP) and one patient deteriorated rapidly and died before receiving chemotherapy. One patient with Burkitt’s lymphoma was treated with an etoposide, prednisone, vincristine, cyclophosphamide, doxorubicin, and rituximab (EPOCH-R) protocol, and the other patient developed septic shock, multiorgan failure, deteriorated rapidly and died before receiving chemotherapy. Patients with PTLD were treated first with reduction of immunosuppression, and chemotherapy using R-CHOP was required for treatment of one patient and rituximab was used in the second one, who developed disseminated intravascular coagulopathy and died.

The median follow-up duration was 22 months (range between 1-78 months) in 14 patients for whom data were available. The one-year overall survival rate was 71% and the 2-year overall survival rate was 43% during follow-up.

## Discussion

Corlu et al^14^ showed that secondary renal involvement in patients with B-cell lymphoproliferative disorders is common. In a study from Saudi Arabia, El-Sharkawy et al^15^ found that 15% of patients scanned for routine staging of pathologically confirmed lymphoma showed CT evidence of renal involvement. Kidney involvements at initial presentation occur in 2.7-6% of lymphomas.^6^

In the English literature, only 3 PRLs have been previously reported from Saudi Arabia and data of those cases were summarized in Table 2. In the records of 2 tertiary hospitals, 17 cases of PRL were identified. The diagnosis was based on a percutaneous renal biopsy in 16 cases and on nephrectomy specimens in one case. In the current study, the most common clinical manifestations included renal masses, hematuria, and flank or pelvic pain. These features can be similar to those of renal cell carcinoma or other renal pathologies. The literature shows that typical symptoms of PRL include flank pain, hematuria, hypertension, edema, acute and chronic kidney injuries, and weight loss. The clinical signs of renal involvement were proteinuria, renal failure, and enlargement of the kidney in radiological evaluation.^7,16^

**Table 2:** - Summary of the primary renal lymphoma cases reported from Saudi Arabia.

References	Age/gender	Site	Clinical presentation	Diagnosis
Omer et al^6^	21/F	Bilateral	Fever, weight loss, abdominal pain, and masses	Non-Hodgkin lymphoma
Hugosson et al^9^	4/M	Right kidney	Abdominal distension, pain, and vomiting	Lymphoblastic lymphoma
Al Mulhim et al^10^	45/F	Right kidney	Right flank pain and hematuria	Diffuse large B-cell lymphoma

F: female, M: male

In the current study, renal function impairment or failure was seen in some patients. Acute kidney injury is a well-known clinical feature of bilateral PRL. This presentation has mainly been described with non-Hodgkin b-cell lymphoma, but it is rarely described in Hodgkin lymphoma and T-cell lymphoma.^17,18^ The acute kidney injury that occurs in PRL can be due to prerenal causes, like poor oral intake; renal causes due to direct tumor infiltration; or postrenal causes like direct compression by the renal tumor.^19^

Unilateral renal involvement is more common than bilateral involvement in this study and in others.^20^ Bilateral involvement was seen in 3 (17.6%) cases in this study compared to 7.9% of the cases in the SEER study.^4^

Patients with diffuse bilateral renal involvement by lymphoma can present with bilateral nephromegaly without acute kidney injury.^21^ Primary renal lymphoma can present as a solitary renal mass, and these situations, it mimics renal cell carcinoma. However, in contrast to renal cell carcinomas (RCC), PRL infiltrates surrounding tissue rather than displacing the kidney, which is usually seen with RCC.^22^

One case in the current series was a child (4 years) who was diagnosed with Burkitt’s lymphoma. Primary renal lymphoma is an extremely rare entity in pediatric age groups. Children with PRL have variable clinical presentation that includes constitutional signs and symptoms, palpable abdominal masses, gross hematuria, anemia, acute kidney injury, and advanced chronic kidney disease.^23-25^ In children, lymphoblastic lymphoma, Burkitt lymphoma were reported in addition to DLBCL.^23-26^

In the current study, radiological evaluation by CT revealed one or more renal hypodense lesions with or without associated local enlarged lymph nodes as the most common features. In 2 cases, only US studies were available, and both had hypoechoic tumors.

The etiology of PRL is not completely understood. Kidneys lack lymphoid tissue, and PRL probably arises from lymphoid cells that arrive at the kidney in different ways. Several suggestions have been reported to explain the pathogenesis, such as an inflammation source, like what happens in the background of chronic inflammation, such as chronic thyroiditis, *Helicobacter pylori* gastritis, and follicular cystitis. This inflammatory process may lead to lymphoma. Chronic pyelonephritis in the kidney may be the source of lymphoid malignancy. Infectious processes are another suggestion in the pathogenesis, as in the association between PRL and EBV infections. Immunocompromised patients have a higher frequency of renal lymphoproliferative diseases. However, the reported cases of PRL in patients with human immunodeficiency virus (HIV) infection are scarce.^19^ Primary renal lymphoma may also originate from the lymphatics surrounding the renal capsule.^4^

Utilizing the SEER database, Taneja et al^2^ and Chen et al^4^ showed that DLBCL is the most common histopathological subtype. Other very rare histological types of PRL that have been described in the literature, including Burkitt, lymphoblastic, lymphocytic, mucosa-associated lymphoid tissue, anaplastic follicular lymphoma, T-cell lymphoma, Hodgkin’s lymphoma, and intravascular large B-cell lymphoma.^7,8,18,24,27-31^ Primary renal pelvis and ureteral lymphomas are extremely rare.^31^ Mucosa-associated lymphoid tissue lymphoma is a relatively common lymphoma in the renal pelvis and ureter. Follicular lymphoma has also been described in the renal pelvis.^31^

Most of the patients received standard chemotherapy for DLBCL with R-CHOP. Early diagnosis and treatment may improve survival of PRL, but the 5-year survival rate is only around 40-50%.^32^ The prognosis of PRL depends mainly on the tumor stage.^19^ Using data from the SEER, Chen et al^4^ demonstrated that the incidence rate of PRL has been increasing significantly. They reported that the 5-year relative survival rate of patients with PRL was 64%.^4^ The one-year overall survival rate in this study was 71% and the 2-year overall survival rate was 43% during follow-up. Typically, nephrectomy is the recommended therapy for renal masses, but nephrectomy for PRL is controversial. Some authors consider nephrectomy as part of the management plan in addition to chemotherapy in unilateral PRL.^1^

Primary renal lymphoma must be differentiated from renal cell carcinoma. Standard treatment of renal masses is nephrectomy, but PRL differs in that it is usually managed with chemotherapy followed by nephrectomy. Therefore, accurate diagnosis of PRL based on renal biopsy is critical to choosing a management plan.^32^ Primary renal lymphoma is mostly misidentified clinically as RCC, but diagnostic imaging may help in differentiation. It is recommended that a biopsy be carried out for renal mass in patients who show features that are not typical for RCC or demonstrate symptoms or signs that are suggestive of a lymphoma such proteinuria or lymphadenopathy.^33^ Primary renal lymphoma can be also misdiagnosed as retroperitoneal hematoma.^34^

The pathological differential diagnosis of PRL depends on the lymphoma type. Renal Burkitt’s lymphoma and lymphoblastic lymphoma must be differentiated from other small, blue, and round-cell tumors (namely, Ewing sarcoma/primitive neuroectodermal tumor, synovial sarcoma, Wilm’s tumor, neuroblastoma, and small cell neuroendocrine tumor). Therefore, immunocytochemistry and fluorescence in situ hybridization (FISH) studies are helpful to establish the diagnosis. All of these conditions can arise in the kidney.

Primary renal lymphoma has been reported in association with other malignancies, including colon cancer and Kaposi sarcoma.^35,36^ It has also been reported in association with other diseases such as chronic hepatitis C infection and Turner syndrome.^16,37^ Synchronous PRL and RCC have been described in the literature.^38-40^

According to the data obtained from SEER, Taneja et al^2^ showed that the median age is above 70 years. Unilateral neoplasm is more commonly detected than bilateral neoplasm, males are more affected than females, and the tumors are seen more commonly in white patients. Diffuse large B-cell lymphoma was the most common histological type. Poor prognostic factors include age ≥60 years and DLBCL histological type. Chen et al^4^ noticed that there was an increased incidence rate of PRL from 1980-2013 in both men and women. Improvement in the overall survival was noticed in the period of 2000-2013 compared to 1980-1999, which was related to the introduction of immunotherapy in addition to chemotherapy.^2^ The pattern of PRL in this study is like the SEER data regarding gender distribution, clinical presentation, and pathological subtypes; however, the median age was lower (50.5 years) in this study, which is younger than that of the SEER data.

### Study limitations

The study covered the PRL in only 2 hospitals and the number of patients is limited. National-wide multicenter studies will be helpful to further explore the disease pattern in Saudi Arabia.

In conclusion, this study shed light on the pattern of PRL in our community. The diagnosis of PRL is important to avoid tumor dissemination and unnecessary nephrectomy. Primary renal lymphomas in Saudi patients are more common in males and seen at relatively younger age group compared to the available SEER data. Diffuse large B-cell lymphoma is the most common pathological type and non-GCB is the most common subtype. The most common clinical presentations are renal mass, flank pain, and hematuria. Bilateral involvement is rare and seen more in Burkitt’s lymphoma than DLBCL.
